# Striped bass (*Morone saxatilis*) migration timing driven by estuary outflow and sea surface temperature in the San Francisco Bay-Delta, California

**DOI:** 10.1038/s41598-020-80517-5

**Published:** 2021-01-15

**Authors:** Pascale Goertler, Brian Mahardja, Ted Sommer

**Affiliations:** 1 California Department of Water Resources, Division of Environmental Services, 3500 Industrial Blvd, West Sacramento, CA 95691 USA; 2 United States Fish and Wildlife Service, 850 S Guild Ave #105, Lodi, CA 95240 USA

**Keywords:** Climate-change ecology, Conservation biology, Freshwater ecology

## Abstract

The influence of climate on the timing of large-scale animal migrations is a major ecological and resource management concern. Anadromous fish migrations can have broad scale impacts on human communities and marine, aquatic and terrestrial food webs. However, isolating the effects of climate change on the timing of life stage transitions for anadromous fish species is challenging. Striped bass (*Morone saxatilis*) exhibit striking variation in migration patterns within their natural range, including migratory behaviors that change with latitude, and climate-induced temperature changes are predicted to drive future habitat and distribution changes. Here we explore the linkages between migration and multiple components of coastal and inland aquatic ecosystems impacted by climate change. By leveraging environmental and fisheries monitoring which began in 1969, we describe the upstream migration timing of non-native adult Striped bass influenced by estuary outflow and sea surface temperature in the San Francisco Bay-Delta, California. Striped bass migrated later in years when Delta outflow was greater and sea surface temperature was cooler. It is likely that temperature thresholds in the ocean during the springtime provide a cue for Striped bass to initiate migration, but sea surface temperature may also represent composite climatic trends influencing Striped bass. Further, the observed variation in migration timing of adult Striped bass has implications for predation risk on the seaward-migration of juvenile Chinook salmon.

## Introduction

The migration of aquatic organisms from coastal to inland habitats represents one of the most conspicuous biological connections between marine and terrestrial environments and has been a major focus of ecological research^1^. These large-scale coordinated movements generally relate to spatially variable resources important for survival or reproduction^2,3^. In western North America, anadromy can facilitate long-distance connectivity via the transfer of marine nutrients to riverine and terrestrial ecosystems^4^. Pacific salmon (*Oncorhynchus *sp.) utilize highly productive marine environments and then following sensory cues, deposit those marine nutrients into their natal streams after returning to spawn^5^. This nutrient and energy transfer in salmon spawning streams has been shown to be profoundly important for both aquatic and terrestrial ecosystems in the Pacific Northwest^6^.

The extent and timing of aquatic migrations can be influenced by multiple biotic and abiotic factors at multiple scales^7^. For example, migration and spawning failure in returning Sockeye salmon (*O. nerka*) has been associated with a genomic signature thought to be linked to a virus infecting fish before river entry^8^, in addition to high river discharge and warm water temperature^9^. Hence, the effects of major changes on migration can be difficult to isolate, making it challenging to predict responses to change. Despite interest in coastal migrations of fish, data is often lacking to evaluate broad scale issues such as climate change. However, long-term data on anadromous migration has provided opportunities for observing rapid changes in migration in response to changes in the environment^10^.

Due to the collapse of key fisheries resources, the San Francisco Bay-Delta watershed is the setting for intensive and long-term aquatic monitoring programs, primarily focused on the ecological role of freshwater flow as an indicator for habitat quality^11–13^. On the Pacific coast of North America, the San Francisco Bay-Delta watershed is inhabited by many species undergoing long distance migrations, most of which are also in significant long-term decline^14^. These species declines are in part due to a heavily modified landscape. Historically, anadromous species had access to a drainage area roughly two-thirds the state of California, but impassable dams reduced available upstream habitat to approximately 5% of the historically available river mileage^15^. Further, the remaining accessible watershed is channelized with approximately 80% of the channels hardened by shoreline armoring and greater than 95% of tidal freshwater wetlands lost or degraded^16^. Given that changes in global climate may compound the effect of habitat loss, there is an urgent need to evaluate species in decline, such as the Striped bass (*Morone saxatilis*) population.

Striped bass exhibit remarkable variation in migration patterns including migratory behaviors that change with latitude^1^, skipped spawning^17^ and divergent migration based on rearing conditions^18^. Striped bass are native to eastern North America and can be found from the St. Lawrence River in Canada to St. John’s River in Florida. In 1879, Striped bass were introduced to the Sacramento-San Joaquin watershed and stocked by the California Department of Fish and Wildlife (CDFW) until 2001^19^. California Striped bass are thought to have quickly distributed themselves along the Pacific coast of North America to other watersheds (such as the Monterey Bay and Russian River) and supported a commercial fishery until 1935^20^. Acoustically tagged striped bass have been observed throughout the San Francisco Bay-Delta watershed^21^ and are thought to spawn in the Sacramento, Feather and San Joaquin rivers^22^. In the California Central Valley Striped bass are both a valuable recreational game fish and predator of threaten native fish. For example, studies have shown that Striped bass predation could have severe impacts on juvenile Chinook salmon (*Oncorhynchus tshawytscha*)^23–25^. California Central Valley Chinook salmon are comprised of four runs, which describe the season in which adults return to their natal freshwater system to spawn: winter, spring, fall and late-fall^26^. Chinook salmon populations have experienced major long-term declines, with both winter and spring run listed under the Endangered Species Act as endangered and threatened, respectively.

In the San Francisco Bay-Delta, surveys primarily targeting juvenile Striped bass have shown a decline since the 1990s concurrent with a number of native and introduced estuarine and pelagic species^27^. Further, climate-induced temperature changes are predicted to drive future habitat and distribution changes for Striped bass^28^. In this study, we examine trends in migration of adult Striped bass in the San Francisco Bay-Delta. By leveraging environmental and fisheries monitoring which began in 1969, we explore the linkages between migration and regional environmental variables impacted by climate change, which have the potential to modify multiple components of coastal and inland aquatic ecosystems. Here, we address how: (1) migration timing has changed over the nearly 50-year time series (1969–2016), and; (2) which environmental variables (spring ocean conditions, estuarine hydrology and temperature) influence these changes in migration.

## Results

### Migration timing

**Figure 1:**
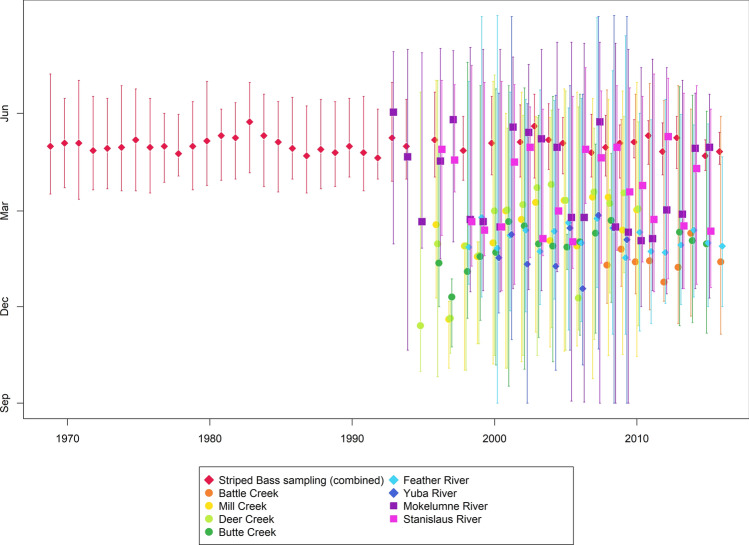
Median migration date for Striped bass and juvenile Chinook salmon. Each species and location sampled is denoted by color, with like areas of juvenile Chinook sampling grouped by symbol: circles for upper tributaries of the Sacramento River, diamonds for locations within the Feather River watershed, and square for watersheds that are not tributaries of the Sacramento River. Median date is represented by a single point and sampling period is represented by a vertical line through that point. The y-axis is September 1st to August 31st.

Striped bass migration timing was variable and has decreased through time (e.g. slope of the linear relationship between median date and year as response and predictor variable, respectively, was negative, but not significant). Median migration timing of Striped bass varied from the fourth week of June (1983) to the third week of May (1987, 1992 and 2015) (Table 1 and Fig. 1).

**Table 1:** A summary of the San Francisco Bay-Delta Interagency Ecological Program’s (IEP) Striped bass Population Study, which captured 307,727 adult Striped bass over the forty-two years examined in this study and exhibited inconsistency in sampling through time. The early (5th percentile), middle (50th percentile) and late (95th percentile) phase of upstream migration of Striped bass for each year was calculated. In these estimates of migration timing, the day of year corresponds to an October 1 to September 30 calendar, which parallels the accumulation of precipitation in California, ecology of juvenile Chinook salmon and is commonly referred to as a “water year”. The cumulative number of individuals captured is provided in parentheses. The sampling inconsistencies are summarized by the gear used and number of locations for each gear in parentheses, median river kilometer (RKm) where fish were captured, and range of capture days (date range of capture data was used as zero data was not provided, and reportedly uncommon).

Year	5th Percentile	50th Percentile	95th Percentile	Gear type (# sites)	Median RKm	Sampling date range
1969	212 (624)	243 (8053)	276 (15,548)	Fyke Trap (2), Gill Net (7)	45	198–311
1970	217 (492)	246 (6616)	270 (13,579)	Fyke Trap (2), Gill Net (3)	52	204–288
1971	217 (878)	246 (8593)	288 (17,186)	Fyke Trap (1), Gill Net (2)	61	193–305
1972	215 (482)	239 (8197)	278 (17,333)	Fyke Trap (1), Gill Net (4)	77	202–290
1973	217 (762)	241 (7458)	266 (14,148)	Fyke Trap (1), Gill Net (1)	79	203–288
1974	216 (612)	242 (6520)	272 (12,943)	Fyke Trap (1), Gill Net (1)	69	201–300
1975	221 (420)	249 (4356)	282 (8391)	Fyke Trap (1), Gill Net (2)	74	201–297
1976	214 (431)	242 (4747)	274 (9983)	Fyke Trap (1), Gill Net (1)	71	199–288
1977	212 (168)	243 (2405)	267 (4643)	Gill Net (1)	42	209–274
1978	216 (157)	236 (2126)	267 (3882)	Gill Net (1)	42	215–273
1979	216 (528)	243 (5318)	271 (10,459)	Fyke Trap (1), Gill Net (1)	56	202–285
1980	216 (286)	248 (3191)	278 (6055)	Fyke Trap (2), Gill Net (1)	69	206–304
1981	218 (279)	253 (3693)	269 (6860)	Fyke Trap (1), Gill Net (2)	72	211–278
1982	219 (151)	251 (1638)	273 (3101)	Fyke Trap (1), Gill Net (2)	64	212–284
1983	219 (144)	266 (1471)	292 (2908)	Fyke Trap (1), Gill Net (2)	47	218–303
1984	215 (216)	253 (2340)	272 (4565)	Fyke Trap (1), Gill Net (2)	77	205–292
1985	216 (244)	247 (3248)	270 (6948)	Fyke Trap (1), Gill Net (2)	53	200–285
1986	218 (237)	241 (3257)	270 (6349)	Fyke Trap (1), Gill Net (3)	56	212–289
1987	212 (240)	234 (3238)	259 (6142)	Fyke Trap (1), Gill Net (4)	53	199–281
1988	217 (278)	240 (3275)	269 (6299)	Fyke Trap (1), Gill Net (5)	61	203–287
1989	217 (197)	237 (3607)	266 (6787)	Fyke Trap (1), Gill Net (4)	45	205–285
1990	217 (337)	243 (3445)	278 (6823)	Fyke Trap (1), Gill Net (4)	64	214–288
1991	215 (373)	237 (4176)	275 (7855)	Fyke Trap (1), Gill Net (4)	58	201–290
1992	214 (216)	232 (2245)	256 (4330)	Fyke Trap (1), Gill Net (3)	42	212–278
1993	217 (250)	251 (3482)	289 (6659)	Fyke Trap (1), Gill Net (5)	108	210–303
1994	216 (168)	243 (3149)	266 (6061)	Fyke Trap (1), Gill Net (5)	64	212–287
1996	221 (359)	249 (3056)	272 (6812)	Fyke Trap (1), Gill Net (4)	42	214–294
1998	217 (236)	239 (2415)	263 (4668)	Gill Net (4)	35	211–271
2000	216 (311)	246 (5482)	272 (10,365)	Fyke Trap (1), Gill Net (4)	42	216–290
2002	219 (292)	247 (3029)	272 (6168)	Gill Net (4)	40	214–282
2003	215 (201)	262 (1968)	277 (3660)	Fyke Trap (1), Gill Net (4)	42	213–292
2004	228 (165)	249 (2033)	277 (3882)	Fyke Trap (1), Gill Net (4)	119	221–283
2005	218 (265)	246 (2871)	265 (5547)	Fyke Trap (1), Gill Net (4)	132	217–270
2007	218 (144)	237 (2559)	255 (5382)	Fyke Trap (1)	171	214–273
2008	219 (88)	242 (2096)	262 (4134)	Fyke Trap (1), Gill Net (2)	100	215–272
2009	217 (82)	246 (1209)	261 (2166)	Fyke Trap (1), Gill Net (2)	118	213–263
2010	232 (148)	247 (968)	262 (1745)	Fyke Trap (1)	171	232–268
2011	230 (244)	253 (2849)	266 (4676)	Fyke Trap (1), Gill Net (1)	171	226–267
2012	223 (323)	238 (2954)	260 (5999)	Fyke Trap (1)	171	216–265
2013	223 (64)	251 (1202)	265 (2351)	Fyke Trap (1)	171	222–271
2015	220 (30)	234 (232)	248 (446)	Fyke Trap (1)	171	220–249
2016	226 (311)	238 (1512)	254 (3557)	Fyke Trap (1)	171	226–256

### Link to environmental variables

To investigate the relative importance of the estuary and coastal ocean on adult Striped bass inland migration, a linear model was employed. Our model tested four central drivers of change in adult Striped bass migration: spring ocean conditions, estuarine hydrology, estuarine temperature and sampling inconsistencies. The model with the lowest AICc score included the slope of upstream movement in sampling location, mean Delta outflow and mean sea surface temperature (Table 2). These three variables were consistent in all top models with the second, third and fourth best models also including median river kilometer, range of days sampled and number of sites sampled, respectively. Delta water temperature was also included in the top five best models (Table 2). The null model was ranked 72nd and the full model ranked 48th. There was little evidence for the influence of centroid day of Delta outflow, Pacific Decadal Oscillation (PDO) or upwelling index.

**Table 2:** Model results for the top fifteen models from lowest to highest AICc. The drivers tested for their influence on median migration date of Striped bass were spring ocean conditions (North Pacific Gyre Oscillation (NPGO), PDO, sea surface temperature, and upwelling index), estuarine hydrology (outflow and centroid day of outflow), estuary temperature, and sampling (upstream movement in sampling location (Slope), median river kilometer (RKm), range of days sampled (Range), and number of sites samples (# sites)). The AICc, AIC weight, adjusted R-squared and p-values are reported.

Model	AICc	wt	R^2^	P-value
1. Sea surface temperature + Outflow + Slope	272.12	0.32	0.36	0.0002
2. Sea surface temperature + Outflow + Slope + RMl	274.78	0.09	0.35	0.0005
3. Sea surface temperature + Outflow + Slope + Range	274.79	0.08	0.35	0.0005
4. Sea surface temperature + Outflow + Slope + # sites	274.85	0.08	0.35	0.0005
5. Sea surface temperature + Outflow + Estuary temperature + Slope	274.85	0.08	0.35	0.0005
6. Sea surface temperature + Outflow	276.84	0.03	0.26	0.0010
7. Sea surface temperature + Outflow + Slope + RMl + Range	277.29	0.02	0.33	0.0013
8. Sea surface temperature + Outflow + Number of sites	277.49	0.02	0.28	0.0016
9. Sea surface temperature + Outflow + Slope + Range + # sites	277.62	0.02	0.33	0.0014
10. Sea surface temperature + Outflow + Slope + RMl + # sites	277.67	0.02	0.33	0.0014
11. Sea surface temperature + Outflow + Estuary temperature + Slope + Range	277.67	0.02	0.33	0.0014
12. Sea surface temperature + Outflow + Estuary temperature + Slope + RMl	277.69	0.02	0.33	0.0014
13. Sea surface temperature + Outflow + Estuary temperature + Slope + # sites	277.74	0.02	0.33	0.0014
14. Outflow + Slope	277.99	0.02	0.24	0.0018
15. Sea surface temperature + Outflow + Range	278.77	0.01	0.25	0.0027

**Figure 2:**
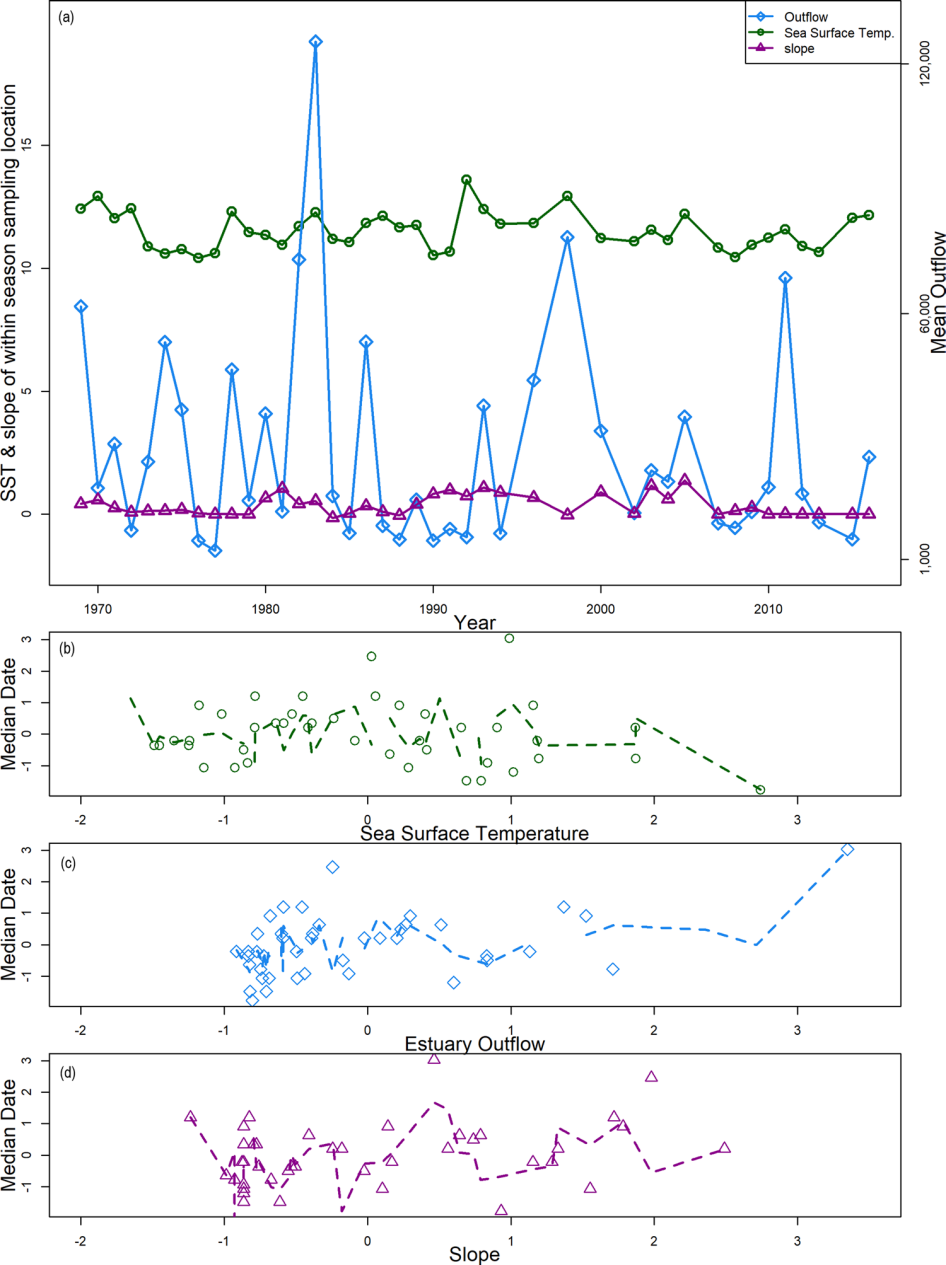
Parameters from the best model in Table 2 plotted through time (**a**) and against median migration date of adult Striped bass (**b**–**d**). Sea surface temperature (°C), mean Delta outflow (cfs), and the slope of within season sampling location are plotted by year (**a**). Then, sea surface temperature (**b**), mean Delta outflow (**c**) and the slope of within season sampling location (**d**) were z-scored and plotted against median date of migration with linear least squares regressions shown by dashed lines. Each variable from the best model in Table 2 is distinguished by color and symbol: sea surface temperature (°C) as green circles, mean Delta outflow (cfs) in blue diamonds, and the slope of within season sampling location denoted by purple triangles.

All regression coefficients in the best model were significant at α = 0.05 (Table S1). Slope and mean outflow were positively related to median date, while the parameter estimate for sea surface temperature was negative. Thus, Striped bass migrated later in years when Delta outflow was greater and sea surface temperature was cooler (Fig. 2).

## Discussion

Increased spring temperature in temperate regions has advanced the seasonal start of reproduction, migration and growth for several migratory species^29,30^. However, for anadromous fish there are relatively few examples of environmentally driven changes in migration timing^10,31^. In California, climate warming is expected to increase both temperature and variability in precipitation^32^, which may have cascading ecosystem impacts. Air temperature in California is projected to increase by 1.5–4.5 °C and sea level rise is expected to be 70–185 cm above the present level^33^. Here we describe variation in migration timing of returning adult Striped bass (*Morone saxatilis*) influenced by estuary outflow and sea surface temperature. As the global climate continues to warm so too may the advance in inland movement of adult Striped bass in the San Francisco Bay-Delta.

**Figure 3:**
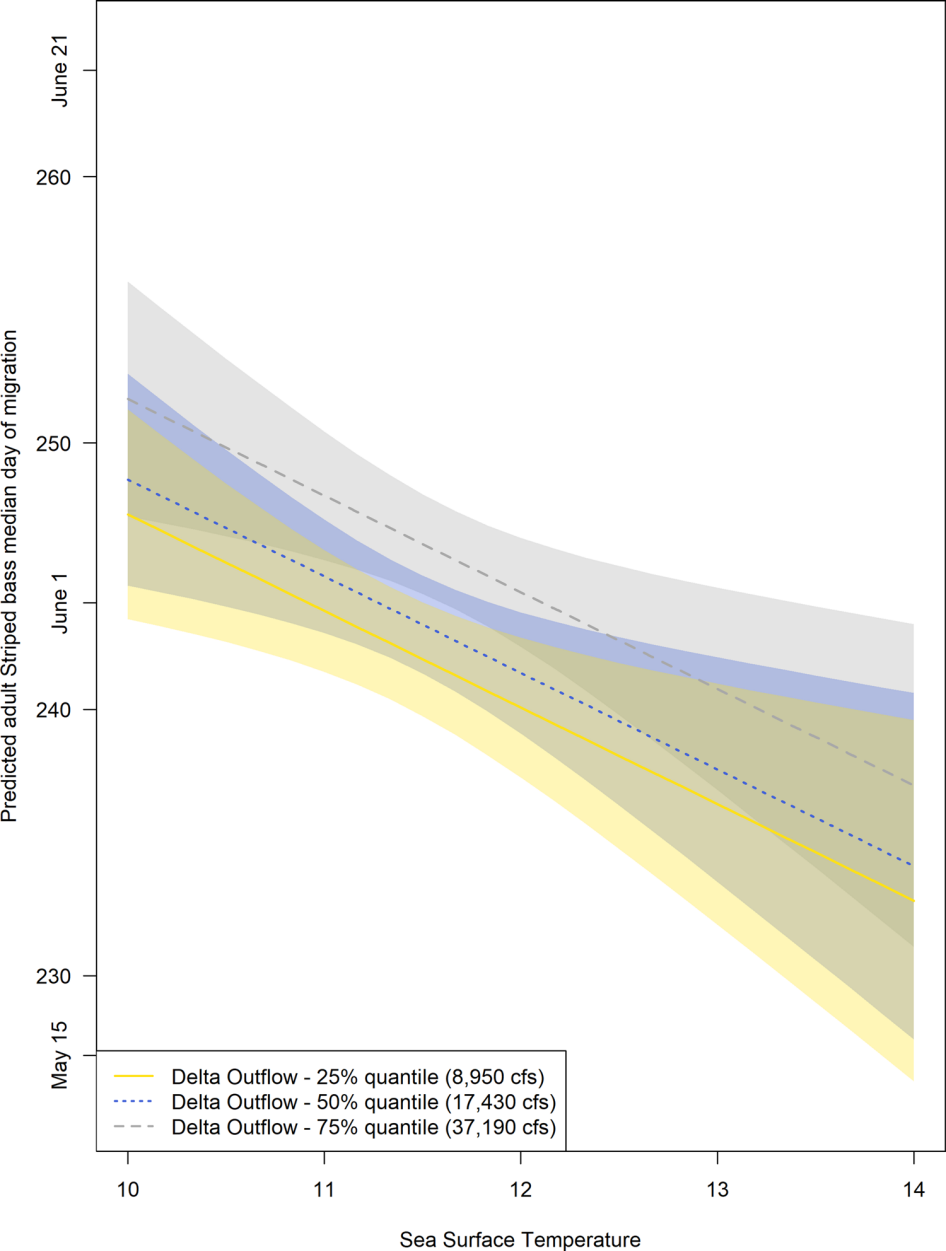
Predicted adult Striped bass median day of migration from the best model, which included the slope of upstream movement in sampling location, mean Delta outflow and mean sea surface temperature (Table S1). The slope of upstream movement in sampling location was held at a mean value of 0.3538, while three values representing the of range Delta outflows described in this study are plotted.

Together these results suggest that increased sea surface temperature congruent with decreased precipitation could shift Striped bass migration earlier in spring (Fig. 3). Studies suggest that animal responses to climate change correspond to seasonality, rather than to temperature^30^. Although this conclusion is complicated by changes in temperature and photoperiod interacting with other environmental conditions, such as food supply^34^. Adult Striped bass were assumed to be on a spawning migration, and therefore could be responding to seasonal cues including, but not limited to temperature. Limited studies exist on the movement patterns of California Striped bass, however their documented migration behaviors during the spawning season are similar to those in their native range^21^.

Environmental conditions in marine habitats could influence the migratory behavior of anadromous fish species in various ways^35,36^. In our study we observed that sea surface temperature was negatively correlated with the timing of Striped bass’ spawning migration (i.e., higher temperatures were associated with earlier migration). This observation is consistent with Striped bass population in its native range in the Chesapeake Bay, where warmer spring water temperature is linked with earlier spawning migration^37^. Although estuary temperature was not included in the best model, the day at which the estuary reached observed spawning temperature for Striped bass (16 °C, 38), decreased across our sampling period, from April 29th in 1969 to April 13th in 2016 (mean = April 20th ± 13 days). It is likely that temperature thresholds in the ocean during the springtime provide a cue for Striped bass to initiate migration, as Striped bass spawning occurs at a specific temperature^39,40^. However, a more complex mechanism is possible given that ocean warming has shifted the phenology of many larval fishes off of the California Coast^41^ and can have a cascading effect in the marine food web^42^. Further, trends in sea surface temperature may represent composite climatic trends influencing Striped bass.

Freshwater inputs are a major driver in estuaries and influence many aspects of the coastal-estuarine ecosystem experienced by Striped bass. Estuary outflow was positively related to median date, indicating that Striped bass migration was delayed when estuary outflow was high (Fig. 2). Striped bass have been shown to hold before moving upriver on flood tides, and this delay could be the result of energetic conservation or osmoregulatory acclimation^1^. Further, the influence of freshwater inputs has been linked to the movements of a suite of adult native species in the San Francisco Bay-Delta^43^. Striped bass are known to display diverse movement patterns, and it has been suggested that this behavioral flexibility may be important for their persistence in this non-native watershed^21^. High freshwater inputs into the estuary provide improved habitat quality for many native fish species^10–12^. Our observed delay in migration may be an indication of Striped bass increased residence time in the estuary in response to similar food web and habitat benefits. Striped bass exhibit both anadromous and freshwater resident life-history strategies, which may have distinct responses to freshwater inputs into the estuary. Further, Striped bass spawning has been observed in the two primary Central Valley rivers (the Sacramento and San Joaquim rivers) as well as the Feather River, a tributary of the Sacramento River^22^. This study did not include sites in the San Joaquin or Feather rivers and therefore may not be accounting for separate populations with distinct behavior.

Variations in the sampling protocol also impacted our estimates of migration timing. When gill netting shifted upstream our estimate of median date increased. Median river kilometer, range of days sampled and the number of sites sampled were also included in the top four models (Table 2). However, these variables are not independent because the two gear-types employed different sampling designs. The inconsistency in sampling complicates our interpretation of these results but is not uncommon given the extent of these data. Long-term fisheries monitoring programs must often navigate year-to-year variation in funding, scope and environmental conditions, which impact sampling effort.

Our results indicate that climate induced changes have already affected the migration timing of the apex aquatic predator in the San Francisco Estuary. It is unclear how earlier arrival at the spawning locations in the Sacramento River might impact Striped bass, but this observed advance in adult Striped bass migration has implications for predation risk on seaward migrating juvenile Chinook salmon. Median migration timing of Striped bass varied from the fourth week of June (1983) to the third week of May (1987, 1992 and 2015) (Table 1). This variation could affect the temporal overlap between juvenile Chinook salmon and adult Striped bass. For example, 89% of the juvenile Chinook salmon examined had exited the river (day of 95% catch, Table S2) by the fourth week of June compared with only 45% by the third week of May. Further, juvenile Chinook migrating from tributaries of the Sacramento River exhibited less overlap in peak migrating timing than those exiting directly into the Delta or lower San Joaquin River (e.g. the Mokelumne and Stanislaus rivers). For example, peak migration of Mokelumne and Stanislaus River salmon regularly coincides with Striped bass (Fig. 1). Although the tributary populations of the Sacramento River exhibit less temporal overlap, climate warming could truncate the seaward-migration period for juvenile Chinook salmon^44^. Striped bass commonly predate on juvenile Chinook salmon with potentially catastrophic consequences. Lindley and Mohr^23^ showed that changes in Striped bass abundance impacted the extinction risk of an endangered Chinook salmon run in California. Further, Striped bass have been shown to capitalize on unnatural structures within migratory routes to predate on salmon^19^. This estuary represents a heavily modified landscape for migratory fish with much of the rivers and tidal freshwater wetlands lost to anadromous species or degraded. Increased temporal overlap with adult Striped bass could heighten the risks navigated by juvenile Chinook as they migrate to the ocean.

Mechanistic studies are needed to quantify the species-scale impact of changes in spring and summer migratory overlap between juvenile Chinook and adult Striped bass. Our results suggest that spring ocean temperature and river discharge are important factors to explore. In addition, we assumed that adult Striped bass were primarily on their spawning migrations as results from acoustically tagged adults indicate increased speeds and river residence in the spring^21^. However, Striped bass also exhibit individual variability in migratory behaviors and estuarine residence, and the life history diversity of this species has not been well-studied in California.

## Materials and methods

### Study system

**Figure 4:**
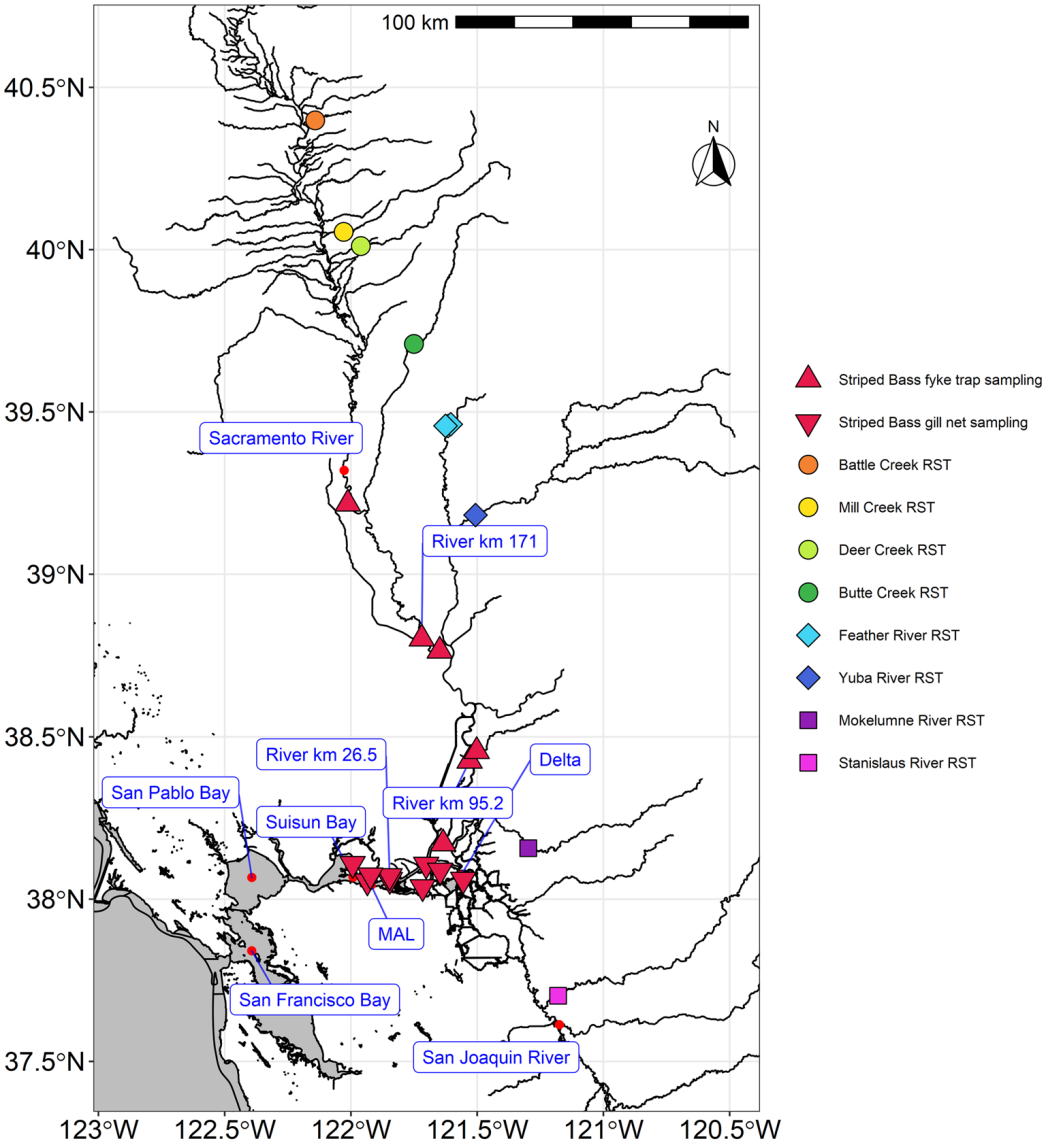
Sampling locations in this study are shown as symbols on the map of the San Francisco Bay-Delta and Central Valley, California. Each species and location sampled is denoted by color, with like areas grouped by symbol: triangles for Striped bass sampling and for the rotary screw trap (RST) sampling of juvenile Chinook salmon, circles denote the upper tributaries of the Sacramento River, diamonds for Feather River watershed, and squares represent the non-Sacramento River tributary watersheds. The map was made using program R version 3.6.3^49^.

Located in California’s Central Valley, the Sacramento and San Joaquin Rivers (and their tributaries) drain into the San Francisco Estuary (SFE). The SFE is made up of a tidal freshwater delta, at the confluence of the Sacramento and San Joaquin rivers (hereafter, the Delta), and a series of large bays increasing in salinity concentration towards the ocean: Suisun, San Pablo and San Francisco bays (Fig. 4). The Sacramento and San Joaquin rivers drain 40% of California’s land area and have been transformed by anthropogenic development, supplying water to 25 million people and four million acres of agriculture^45^.

### Environmental data collection

To test the influence of estuarine and coastal ocean conditions on Striped bass we summarized annual time series for a set of environmental factors assumed to be important to anadromous fish species on the Pacific coast of North America (Table 3). The main effects pertaining to environmental conditions in the estuary and coastal ocean were NPGO, PDO, sea surface temperature, upwelling index, mean Delta outflow, timing of peak Delta outflow, and mean Delta water temperature (Table 3). To estimate the influence of the IEP Striped bass Population Study’s sampling design on Striped bass migration timing four additional main effects were included: the yearly median river kilometer of sampling location (measured from the starting point of the Benicia Bridge, California), the upstream movement in sampling location, the range of days sampled each year and the number of sites sampled each year (Table 3). Summary data was calculated for the Striped bass migration period seen in this study: Julian day 65–189 for daily data, and March to June for monthly data.

**Table 3:** Data sources for the main effects relating to the environmental conditions in the estuary and coastal ocean as well as the sampling design of the IEP Striped bass Population Study, which were then tested for influences on Striped bass yearly median migration date.

Influences on migration timing	Data summarized by year	Location Sampled	Sampling Day Range	Source
Spring ocean conditions	NPGO, PDO, Mean Sea Surface Temperature (°C), Upwelling Index	North Pacific Ocean, Farallon Islands, 39 N 125 W	March to June 65–189	Georgia Institute of Technology^1^, NOAA^2^, Shore Stations Program^3^, NOAA^4^
Hydrology	Mean Delta outflow (cfs), and centroid day of outflow	San Francisco Estuary	65–189	Dayflow^5^
Temperature	Mean Delta water temperature (°C)	Mallard Island	65–189	CDWR^6^, Wagner et al.^48^
Striped bass sampling	Upstream movement in sampling location, median river kilometer of sampling location, range of days sampled, number of sites sampled	All Striped bass sampling locations included (Fig. 1)	65–189	CDFW

^1^https://www.o3-d.org/npgo/npgo.php; accessed 13 September 2017.

^2^https://jisao.washington.edu/pdo/; accessed 12 September 2017.

^3^https://shorestation.ucsd.edu/index.html; accessed 12 September 2017.

^4^https://www.pfeg.noaa.gov/products/PFEL/modeled/indices/upwelling/NA/data_download.html; accessed 12 September 2017.

^5^https://water.ca.gov/Programs/Environmental-Services/Compliance-Monitoring-And-Assessment/Dayflow-Data; accessed 12 May 2017.

^6^https://cdec.water.ca.gov/dynamicapp/staMeta?station_id=MAL.

At the interface between the rivers of the California Central Valley and the Pacific Ocean, these drivers were selected to describe both the aquatic conditions experienced by Striped bass and climate variability impacting anadromous species. For example, the NPGO is a climate pattern which has been linked to fluctuations in nutrient and phytoplankton concentrations^46^, while the PDO describes interdecadal changes in Pacific climate characterized by distinct patterns in sea surface temperature, sea level pressure and the direction and intensity of surface wind stress^47^. Ocean condition were described by NPGO from the Georgia Institute of Technology, PDO from the University of Washington, mean sea surface temperature (°C) measured at the Farallon Islands from the Scripps Institution of Oceanography Shore Stations Program, and monthly upwelling index anomalies at 39° N from the National Oceanic and Atmospheric Administration’s Pacific Fisheries Environmental Laboratory (Table 3). SFE freshwater outflow adjusted for water exports (cfs) was acquired from the California Department of Water Resources (CDWR). Timing of peak outflow was estimated by calculating the centroid of outflow distribution based on Julian day^26^. Delta water temperature (°C) was acquired from a continuous water quality monitoring station managed by CDWR near Mallard Island (38°02′34.5″N 121°55′12.1″W, Fig. 4). Missing water temperature data was estimated using hindcasted daily values in Wagner et al.^48^, which includes water temperature estimates before 1984 (installation of the station) and up to eighty-two days of missing data a year between 1984 and 2016.

### Striped bass data collection

The IEP Striped bass Population Study captured 307,727 adult Striped bass over the 42 years examined in this study and exhibited inconsistency in sampling through time (Table 1). For example, the sampling period duration varied year-to-year and between one and nine sites were sampled each year by gill net and fyke trap (Table 1). Gill nets were primarily used in estuarine sites, while fyke traps were used in riverine sites (Fig. 1). As a result, the gear used was governed by sampling location, which varied within season and year-to-year. Sampling effort intensity was not consistently recorded, and it was assumed that the choice of methods employed maximized the capture of adult Striped bass for that site.

Migration timing of adult Striped bass was estimated by the day at which 5%, 50% and 95% of individuals had been captured each year (Table 1). The day of year corresponds to an October 1 to September 30 calendar, which parallels the accumulation of precipitation in California, ecology of juvenile Chinook salmon and is commonly referred to as a “water year”.

### Data analysis

To investigate the relative importance of the estuary and coastal ocean on adult Striped bass inland migration, a linear model was employed using program R version 3.6.3^49^. Our response variable, Striped bass migration timing, was estimated by the day at which 50% of individuals had been captured each water year (median date). This metric is a commonly used estimate of migration timing for anadromous fish^50,51^. Data diagnostics were assessed following the methods reported in Zuur et al.^52^. Our response variable, median date was normally distributed and independent from time (e.g. no temporal autocorrelation was detected).

In some instances, the IEP Striped bass Population Study’s gill netting followed Striped bass as they migrated upstream (e.g. the sampling location changed within year). To capture the within-season change in sampling location we calculated the slope in sampling location each year (slope of the linear relationship between sampling location and day as response and predictor variable, respectively). No outliers were detected, but there was heterogeneity of variance present for the number of sites sampled each year. The variation inflation factor was less than three for all covariates included in the final models, and so collinearity was not accounted for. Main effects were included as explanatory variables in the linear model, and no interactions were included in the interest of tractability and ease of interpreting results.

The models were compared using Akaike information criterion with a correction for small sample sizes (AICc), using the package MuMIn^53,54^. The model with the lowest AICc value was considered the best representation of the data. Akaike weight was also calculated to give the overall weight of evidence for each model^53^.

### Chinook salmon data collection

To better understand the implications of changes in adult Striped bass migration timing on the predation risk of seaward migrating juvenile Chinook salmon we also included data from eight rotary screw traps. The juvenile Chinook salmon rotary screw trap datasets included in this study ranged from ten (2007–2016 in Battle Creek) to 23 years (1993–2015 in the Mokelumne River) of consecutive sampling (Table S2). The range of days sampled varied from year-to-year and was dependent upon the target species or run for each monitoring program. For example, when steelhead (*O. mykiss*) was the target species the program yielded year-round capture data, while programs primarily targeting Chinook salmon did not sample during summer months. Between one and three traps were deployed on each river, which varied across years. Deer, Mill and Battle Creek deployed a single trap, while Butte (1–2), Yuba (1–3), Mokelumne (1–3), Stanislaus (2–3) and Feather River (2–3) deployed multiple traps. Further, the Mokelumne River changed trap locations up to five times and Feather River up to seven times over the period summarized here. Finally, we intentionally excluded juvenile Chinook salmon data from mainstem trapping locations because of concerns that the unnatural timing of hatchery releases would confound our estimates. Although hatchery marking does occur in the San Francisco Bay-Delta watershed, fractional marking practices make many hatchery-origin fish indistinguishable from wild-origin individuals.

This study synthesizes monitoring data from several sources throughout the California Central Valley. Each dataset was collected in accordance with that monitoring program’s relevant guidelines and regulations. There was no care or use of experimental animals for this study.

## Supplementary Information

Supplementary Tables.
