# Supplementary material for: Striped bass (Morone saxatilis) migration timing driven by estuary outflow and sea surface temperature in the San Francisco Bay-Delta, California
Source: Sci Rep. 2021 Jan 15;11:1510. doi: 10.1038/s41598-020-80517-5 (PMC7810903; doi:10.1038/s41598-020-80517-5)
Supplement: Supplementary file 1 — Supplementary Tables. [file 41598_2020_80517_MOESM1_ESM.docx]

**Striped bass (*Morone saxatilis*) migration timing driven by estuary outflow and sea surface temperature in the San Francisco Bay-Delta, California**

Pascale Goertler^*1^, Brian Mahardja^2^, Ted Sommer^1^

1: California Department of Water Resources, Division of Environmental Services, 3500 Industrial Blvd, West Sacramento CA, 95691

2: United States Fish and Wildlife Service, 850 S Guild Ave #105, Lodi, CA, 95240

*pascale.goertler@deltacouncil.ca.gov

**Supplemental Table 1**: Standardized parameter coefficients and significance in best model.

| Coefficients | Estimate | Std. Error | P-value |
| --- | --- | --- | --- |
| Intercept | 0.0496 | 0.124 | 0.690803 |
| Slope | 0.3403 | 0.127 | 0.010642 |
| Sea Surface Temperature | -0.3840 | 0.132 | 0.005998 |
| Mean Outflow | 0.6207 | 0.149 | 0.000169 |

**Supplemental Table 2**: The early (5^th^ percentile), middle (50^th^ percentile) and late (95^th^ percentile) phase of outmigration for each year and tributary. The migration window is estimated by the day of outmigration (the day of year corresponds to an October 1 to September 30 calendar, which parallels the accumulation of precipitation in California, ecology of juvenile Chinook salmon and is commonly referred to as a “water year”.) and also includes the cumulative number of individuals captured in parentheses, with the day range of sampling in that year and location. Source data can be found at <https://www.fws.gov/cno/fisheries/camp/Documents-Reports/>

|  | Year | 5^th^ Percentile | 50^th^ Percentile | 95^th^ Percentile | Sampling Date Range |
| --- | --- | --- | --- | --- | --- |
| Battle Creek | 2008 | 121 (2,086) | 131 (33,358) | 148 (64,197) | 95-296 |
|  | 2009 | 135 (1,069) | 146 (1,974) | 146 (1,974) | 111-268 |
|  | 2010 | 133 (15,004) | 134 (183,393) | 139 (1,051,462) | 104-292 |
|  | 2011 | 110 (821) | 135 (4,822) | 259 (13,806) | 89-302 |
|  | 2012 | 115 (475) | 115 (475) | 141 (231,324) | 97-286 |
|  | 2013 | 107 (467) | 129 (9,589) | 143 (16,766) | 76-300 |
|  | 2014 | 119 (803) | 161 (24,352) | 161 (24,352) | 83-278 |
|  | 2016 | 126 (888) | 134 (11,136) | 135 (37,304) | 66-271 |
| Mill Creek | 1996 | 156 (5) | 169 (86) | 256 (163) | 100-305 |
|  | 1997 | 64 (2) | 80 (6) | 95 (10) | 34-97 |
|  | 1998 | 145 (2) | 149 (18) | 152 (43) | 32-153 |
|  | 1999 | 133 (56) | 139 (536) | 143 (1,155) | 57-153 |
|  | 2000 | 133 (48) | 152 (913) | 262 (1,755) | 38-304 |
|  | 2001 | 112 (173) | 182 (1,735) | 258 (3,316) | 34-274 |
|  | 2002 | 82 (633) | 174 (6,662) | 271 (12,981) | 40-294 |
|  | 2003 | 69 (56) | 190 (468) | 264 (936) | 68-271 |
|  | 2004 | 120 (151) | 154 (2,190) | 168 (4,225) | 54-270 |
|  | 2005 | 150 (621) | 192 (6,543) | 249 (13,419) | 50-297 |
|  | 2006 | 58 (19) | 149 (214) | 265 (441) | 55-294 |
|  | 2007 | 161 (515) | 195 (6,416) | 257 (12,249) | 24-288 |
|  | 2008 | 49 (35) | 195 (3,732) | 249 (7,187) | 46-287 |
|  | 2009 | 63 (166) | 164 (235) | 264 (444) | 47-295 |
|  | 2010 | 139 (151) | 183 (1,503) | 270 (2,873) | 45-303 |
| Deer Creek | 1995 | 70 (54) | 74 (562) | 173 (1092) | 31-294 |
|  | 1996 | 122 (278) | 151 (2,764) | 237 (5345) | 26-305 |
|  | 1997 | 80 (15) | 81 (53) | 95 (113) | 48-97 |
|  | 1998 | 131 (11) | 149 (90) | 151 (151) | 32-153 |
|  | 1999 | 107 (342) | 140 (2,165) | 148 (6674) | 57-153 |
|  | 2000 | 142 (262) | 182 (3,126) | 242 (6130) | 46-310 |
|  | 2001 | 59 (106) | 183 (1,172) | 249 (2269) | 34-260 |
|  | 2002 | 89 (445) | 188 (4366) | 243 (8527) | 46-272 |
|  | 2003 | 80 (137) | 204 (1427) | 260 (2730) | 46-270 |
|  | 2004 | 139 (476) | 207 (4263) | 256 (9067) | 72-277 |
|  | 2005 | 150 (459) | 192 (5444) | 254 (10733) | 51-295 |
|  | 2006 | 70 (41) | 100 (148) | 266 (264) | 70-280 |
|  | 2007 | 161 (330) | 200 (12836) | 246 (27309) | 61-260 |
|  | 2008 | 125 (20) | 189 (581) | 196 (986) | 105-202 |
|  | 2009 | 145 (11) | 199 (121) | 222 (282) | 124-271 |
|  | 2010 | 135 (61) | 184 (711) | 255 (1383) | 74-266 |
| Butte Creek | 1996 | 103 (3,795) | 133 (38,885) | 153 (74,574) | 92-242 |
|  | 1997 | 84 (37) | 101 (320) | 115 (677) | 54-118 |
|  | 1998 | 121 (323) | 125 (3,196) | 218 (6,151) | 81-322 |
|  | 1999 | 109 (10,743) | 139 (66,828) | 169 (204,588) | 79-300 |
|  | 2000 | 133 (6,613) | 143 (66,694) | 163 (119,183) | 37-297 |
|  | 2001 | 135 (24,658) | 172 (175,630) | 257 (474,370) | 17-300 |
|  | 2002 | 119 (11,096) | 168 (124,532) | 253 (236,615) | 33-298 |
|  | 2003 | 124 (1,340) | 151 (14,647) | 245 (28,240) | 63-272 |
|  | 2004 | 125 (10,489) | 149 (70,686) | 209 (203,655) | 40-290 |
|  | 2005 | 132 (19,108) | 148 (169,897) | 148 (169,897) | 127-160 |
|  | 2006 | 95 (978) | 153 (9,904) | 261 (19,446) | 65-292 |
|  | 2007 | 126 (5,954) | 161 (140,197) | 189 (303,757) | 67-245 |
|  | 2008 | 151 (9,466) | 173 (73,733) | 200 (183,521) | 96-236 |
|  | 2013 | 141 (2,564) | 162 (27,695) | 187 (51,218) | 74-299 |
|  | 2014 | 136 (7,122) | 154 (84,745) | 191 (166,456) | 77-291 |
|  | 2015 | 112 (1,067) | 151 (10,202) | 185 (22,376) | 67-276 |
| Feather River | 1998 | 131 (16,528) | 148 (154,661) | 186 (322,705) | 113-304 |
|  | 1999 | 141 (22,864) | 176 (233,523) | 207 (451,037) | 101-365 |
|  | 2000 | 118 (19,924) | 147 (243,654) | 170 (471,432) | 1-366 |
|  | 2001 | 130 (34,379) | 159 (338,485) | 187 (653,236) | 88-294 |
|  | 2002 | 130 (38,207) | 164 (407,243) | 188 (777,631) | 87-287 |
|  | 2003 | 112 (23,977) | 144 (326,858) | 174 (634,767) | 94-296 |
|  | 2004 | 125 (68,377) | 163 (677,327) | 199 (1,343,111) | 99-286 |
|  | 2005 | 121 (18,361) | 171 (245,666) | 195 (467,733) | 70-279 |
|  | 2006 | 107 (4,061) | 152 (41,604) | 194 (78,759) | 78-288 |
|  | 2007 | 118 (13,578) | 175 (238,428) | 212 (452,589) | 110-364 |
|  | 2008 | 109 (28,774) | 166 (320,149) | 205 (609,456) | 66-314 |
|  | 2009 | 107 (21,299) | 138 (259,786) | 195 (501,034) | 66-347 |
|  | 2010 | 107 (13,298) | 162 (143,857) | 206 (278,946) | 69-274 |
|  | 2011 | 120 (91,111) | 144 (909,130) | 174 (1,736,046) | 76-268 |
|  | 2012 | 118 (50,828) | 143 (506,262) | 170 (1,045,540) | 95-287 |
|  | 2013 | 118 (37,099) | 150 (451,701) | 174 (885,169) | 94-273 |
|  | 2014 | 134 (54,716) | 164 (547,574) | 181 (1,036,334) | 101-263 |
|  | 2015 | 121 (21,708) | 152 (222,177) | 188 (439,263) | 92-264 |
|  | 2016 | 120 (16,438) | 149 (154,235) | 177 (311,359) | 92-233 |
| Yuba  River | 2000 | 107 (15,138) | 138 (151,357) | 165 (376,784) | 86-293 |
|  | 2001 | 122 (27,377) | 160 (275,928) | 198 (526,178) | 61-365 |
|  | 2002 | 101 (13,893) | 132 (189,467) | 204 (365,188) | 1-283 |
|  | 2004 | 98 (5,228) | 130 (152,135) | 190 (293,003) | 32-291 |
|  | 2005 | 114 (12,899) | 166 (143,250) | 208 (273,018) | 51-300 |
|  | 2006 | 92 (1,796) | 109 (24,654) | 112 (51,720) | 37-292 |
|  | 2007 | 122 (19,606) | 178 (190,756) | 241 (386,148) | 52-365 |
|  | 2008 | 112 (10,562) | 166 (124,034) | 244 (236,950) | 1-366 |
|  | 2009 | 116 (3,172) | 155 (36,601) | 240 (69,728) | 1-365 |
| Mokelumne  River | 1993 | 171 (2,171) | 275 (22,462) | 301 (42,665) | 151-332 |
|  | 1994 | 64 (739) | 233 (8,367) | 305 (15,989) | 51-334 |
|  | 1995 | 153 (999) | 172 (11,226) | 278 (21,800) | 147-331 |
|  | 1996 | 154 (292) | 229 (7,437) | 286 (14,184) | 137-335 |
|  | 1997 | 241 (5,657) | 268 (55,731) | 297 (109,966) | 153-333 |
|  | 1998 | 141 (5,666) | 174 (63,299) | 281 (120,315) | 106-336 |
|  | 1999 | 154 (1,029) | 172 (33,445) | 275 (64,171) | 107-334 |
|  | 2000 | 148 (121) | 167 (7,468) | 281 (14,158) | 106-334 |
|  | 2001 | 166 (2,994) | 261 (34,343) | 291 (67,191) | 109-327 |
|  | 2002 | 160 (872) | 256 (8,811) | 291 (17,641) | 108-320 |
|  | 2003 | 163 (532) | 250 (4,844) | 282 (10,172) | 107-334 |
|  | 2004 | 155 (217) | 242 (2,249) | 339 (4,253) | 125-341 |
|  | 2005 | 4 (175) | 176 (3,880) | 308 (7,527) | 3-341 |
|  | 2006 | 62 (974) | 176 (11,525) | 336 (19,498) | 2-340 |
|  | 2007 | 2 (625) | 266 (11,035) | 339 (20,790) | 1-341 |
|  | 2008 | 5 (41) | 167 (7,314) | 279 (15,854) | 1-340 |
|  | 2009 | 62 (136) | 162 (2,020) | 237 (4,248) | 1-334 |
|  | 2010 | 128 (399) | 154 (4,488) | 266 (8,763) | 92-317 |
|  | 2011 | 141 (1,440) | 156 (15,213) | 191 (31,921) | 107-325 |
|  | 2012 | 140 (884) | 183 (11,127) | 278 (21,222) | 104-317 |
|  | 2013 | 139 (4,523) | 179 (46,831) | 272 (88,969) | 109-305 |
|  | 2014 | 145 (2,958) | 241 (33,209) | 264 (63,390) | 109-293 |
|  | 2015 | 150 (3,052) | 242 (30,899) | 262 (59,522) | 100-292 |
| Stanislaus River | 1996 | 159 (89) | 240 (1,126) | 269 (2,341) | 159-305 |
|  | 1997 | 202 (76) | 230 (1,168) | 270 (2,215) | 200-301 |
|  | 1998 | 151 (811) | 172 (9,728) | 259 (18,496) | 130-319 |
|  | 1999 | 143 (1,747) | 164 (20,582) | 243 (39,144) | 140-303 |
|  | 2000 | 149 (3,305) | 167 (35,567) | 224 (69,938) | 107-304 |
|  | 2001 | 187 (452) | 228 (4,955) | 264 (9,383) | 113-301 |
|  | 2002 | 179 (170) | 242 (1,909) | 257 (3,625) | 138-280 |
|  | 2003 | 151 (4,458) | 156 (6,997) | 249 (13,352) | 140-278 |
|  | 2004 | 173 (1,176) | 182 (19,124) | 233 (38,038) | 132-278 |
|  | 2005 | 151 (720) | 153 (12,267) | 233 (24,005) | 128-290 |
|  | 2006 | 190 (71) | 240 (792) | 285 (1,498) | 189-317 |
|  | 2007 | 180 (143) | 232 (1,255) | 276 (2,751) | 133-295 |
|  | 2008 | 151 (11) | 242 (102) | 271 (217) | 145-300 |
|  | 2009 | 189 (38) | 200 (374) | 258 (720) | 136-298 |
|  | 2010 | 145 (45) | 206 (535) | 263 (1,037) | 134-290 |
|  | 2011 | 125 (22) | 174 (237) | 273 (575) | 106-311 |
|  | 2012 | 157 (54) | 252 (565) | 274 (1,136) | 118-307 |
|  | 2013 | 154 (540) | 168 (9,183) | 257 (17,894) | 109-293 |
|  | 2014 | 139 (66) | 222 (1,041) | 265 (1,959) | 113-300 |
|  | 2015 | 163 (64) | 163 (64) | 221 (844) | 110-278 |
